# Safe Cultivation of *Medicago sativa* in Metal-Polluted Soils from Semi-Arid Regions Assisted by Heat- and Metallo-Resistant PGPR

**DOI:** 10.3390/microorganisms7070212

**Published:** 2019-07-22

**Authors:** Anas Raklami, Khalid Oufdou, Abdel-Ilah Tahiri, Enrique Mateos-Naranjo, Salvadora Navarro-Torre, Ignacio D. Rodríguez-Llorente, Abdelilah Meddich, Susana Redondo-Gómez, Eloísa Pajuelo

**Affiliations:** 1 Laboratory of Biology and Biotechnology of Microorganisms, Faculty of Sciences Semlalia, Cadi Ayyad University, PO Box 2390, Marrakech, Morocco; 2 Laboratory of Biotechnology and Plant Physiology, Faculty of Sciences Semlalia, Cadi Ayyad University, PO Box 2390, Marrakech, Morocco; 3 Department of Microbiology and Parasitology, University of Seville, PO Box 1095, 41080 Seville, Spain; 4 Department of Plant Biology and Ecology, University of Seville, PO Box 1095, 41080 Seville, Spain

**Keywords:** heavy metal stress, PGPR, rhizobia, legumes, stress related genes, ROS-scavenging enzymes, biofilms, scanning electron microscopy

## Abstract

Soil contamination with heavy metals is a constraint for plant establishment and development for which phytoremediation may be a solution, since rhizobacteria may alleviate plant stress under these conditions. A greenhouse experiment was conducted to elucidate the effect of toxic metals on growth, the activities of ROS (reactive oxygen species)-scavenging enzymes, and gene expression of *Medicago sativa* grown under different metal and/or inoculation treatments. The results showed that, besides reducing biomass, heavy metals negatively affected physiological parameters such as chlorophyll fluorescence and gas exchange, while increasing ROS-scavenging enzyme activities. Inoculation of *M. sativa* with a bacterial consortium of heat- and metallo-resistant bacteria alleviated metal stress, as deduced from the improvement of growth, lower levels of antioxidant enzymes, and increased physiological parameters. The bacteria were able to effectively colonize and form biofilms onto the roots of plants cultivated in the presence of metals, as observed by scanning electron microscopy. Results also evidenced the important role of glutathione reductase (*GR*), phytochelatin synthase (*PCS*), and metal transporter *NRAMP1* genes as pathways for metal stress management, whereas the gene coding for cytochrome P450 (*CP450*) seemed to be regulated by the presence of the bacteria. These outcomes showed that the interaction of metal-resistant rhizobacteria/legumes can be used as an instrument to remediate metal-contaminated soils, while cultivation of inoculated legumes on these soils is still safe for animal grazing, since inoculation with bacteria diminished the concentrations of heavy metals accumulated in the aboveground parts of the plants to below toxic levels.

## 1. Introduction

According to FAO, which declared the past year 2015 as the International Year of the Soils [1], the pressure of the global human population, together with the increasing demand for food, feed, biodiesel, wood, fibers, etc. will force farmers to cultivate plants on poor, degraded, or moderately-polluted soils [2,3]. Two aspects of the interaction between metals and plants arise when considering cultivation of plants on metal polluted soils: on the one hand, phytoremediation of soils and, on the other hand, a concern for metal accumulation on plant tissues and its subsequent distribution to the trophic web [4,5]. In particular, some medicinal plants have been proposed to be grown on polluted soils [6,7], since they behave as metal excluders [8,9], and the low concentrations of metals accumulated in shoots do not affect the ulterior quality of extracted oils or bioactive substances [10,11,12].

In this context, legumes are also optimum candidates to adapt to degraded soils, particularly to those affected by moderate heavy metal pollution. Legumes are among the plants that have been proposed for phytoremediation [13,14,15,16]. These plants behave as metal excluders, since they mainly accumulate metals in the roots and display low translocation to the aboveground part of plants [13,17]. In addition, legumes are known for their ability to associate with plant growth-promoting bacteria (PGPR) and rhizobia that generate a biological fertilization of soils that allows the ultimate installation of other plant species and microorganisms [18,19]. These microorganisms can provide nutrients to plants and reduce the adverse effects of contaminants on the plant [20,21]. In this way, plant growth-promoting bacteria (PGPR) and rhizobia-type bacteria associated with leguminous plants used for phytoremediation can contribute to the tolerance, growth, absorption, and acceleration of the process [14,22,23]. Beneficious rhizobacteria improve plant growth by various direct and indirect mechanisms, particularly including PGP activities such as phosphate solubilization, phytohormone production, and deamination of aminocyclopropane carboxylic acid (CCA), the precursor of ethylene, etc. [24,25]. Through these mechanisms, the health and the robustness of plants are enhanced, facilitating the adaptation of plants under stressful conditions [26,27]. Furthermore, these bacteria can contribute to the process of phytoremediation, through mechanisms including improved solubilization of metals, siderophore production, production of organic acids and biosurfactants, reduction/oxidation, methylation, precipitation, and biosorption that affect the bioavailability of metals in soils and sediments [22,28,29].

In order to counteract metal stress, plants have evolved multiple extracellular and intracellular mechanisms to tolerate and regulate the uptake of heavy metals. The extracellular mechanisms are manifested by the establishment of symbiotic interactions with bacteria and/or mycorrhizal fungi. [26,30] At the intracellular scale, stress tolerance is associated with the modification of membrane permeability, restriction in water (and metal) uptake, and complexation or immobilization in the rhizosphere, etc. [31,32]. Once inside the cell, heavy metals induce oxidative, osmotic, and ionic stresses in plants [33,34], which are accompanied by the induction of antioxidant enzymes such as superoxide dismutase (SOD), ascorbate peroxidase (APX), glutathione peroxidase (GR), catalase (CAT), and low molecular weight deactivating molecules such as ascorbic acid and thiols such as glutathione and proline. Moreover, the intracellular presence of organic ligands, namely metallothionins, phytochelatins, organic acids, and amino acids, ensures complexation and therefore the detoxification of metal ions [35,36,37]. From the point of view of omics, adaptation to metals implies the overexpression of multiple genes belonging to different pathways such as cysteine synthase, glutathione transferase, and glutathione reductase for the metabolism of glutathione and phytochelatins, genes of secondary metabolism such as phenylalanine ammonia lyase, and genes involved in drought stress such as the proline synthesis pathway, metal transporters, etc. [34,38,39]. Therefore, studies are needed to understand the plant–bacteria relationship and to identify genes and pathways that participate in the management of metal stress.

Accordingly, the present study was conducted with the following objectives: (1) to evaluate the consequence of inoculation with previously selected PGPR on the growth, mineral nutrition, and physiological responses of *Medicago sativa* to heavy metal stress; (2) to evaluate the effect of PGPR on heavy metal uptake; and (3) to analyze the effect on the activities of ROS-scavenging enzymes and expression level of marker genes belonging to different pathways related to stress management.

## 2. Materials and Methods

### 2.1. Plant Growth Conditions and Inoculation Treatments

*M. sativa* seeds were surface disinfected with commercial sodium hypochlorite solution diluted 1/5 (v/v) for 5 min. After several rinsing with distilled water, seeds were placed on wet filter paper in petri dishes for germination in the dark at 28 °C for 24 h. The germinated seeds were sown in plastic pots (2 L capacity) filled with previously sterilized natural compost, sand, and perlite at 1:1:1 ratio (w/w). The pots were watered with water either supplemented or not with heavy metals at two different concentrations: high metal concentrations (HMC) and low metal concentrations (LMC). HMC corresponded to the following metal concentrations: Cu: 2.30 mM, Pb: 0.35 mM and Zn: 4.30 mM. LMC corresponded to the following metal concentrations: Cu: 1.15 mM, Pb: 0.18 mM and Zn: 2.15 mM. These concentrations were selected based on the levels of the abovementioned metals found in the Kettara mine, Marrakesh, Morocco [40]. The pots were divided into two different inoculation treatments (inoculated and non-inoculated) with a bacterial consortium composed of four metallo-tolerant strains isolated from metal-polluted soils of the semi-arid region of Marrakech, i.e., *Proteus* sp. DSP1, *Pseudomonas* sp. DSP17, *Ensifer meliloti* RhOL6 and RhOL8. These bacterial strains were isolated from metal polluted soils of the Kettara mine and display, simultaneously, several plant growth-promoting traits (phosphate solubilization, nitrogen fixation, secretion of siderophores, and production of indolacetic acid), besides displaying a high tolerance towards heavy metals (Cu, Pb, and Zn) and high temperatures (Raklami et al., accompanying paper). The rhizobial strains RhLO6 and RhLO8 were grown overnight in yeast extract-mannitol (YEM) medium [41] at 28 °C with continuous shaking at 140 rpm. The PGPR strains DSP1 and DSP17 were cultivated in tryptone soy (TSB) medium at 28 °C with continuous shaking at 140 rpm. The absorbance at 600 nm of the cultures was adjusted to 1.0 and equal volumes of the four strains were mixed. Every pot was inoculated with 10 mL of the bacterial consortium at the beginning of the experiment and once a week during the first month. The pots were placed in individual trays in the greenhouse of the Centre for Research, Technology and Innovation of the University of Sevilla, Spain (CITIUS) with a controlled temperature between 21 °C and 25 °C, 40–60% relative humidity, and natural daylight of 250 µmol m^−2^ s^−1^ as a minimum and 1000 µmol m^−2^ s^−1^ as a maximum during spring of 2018. Watering was done with 250 mL of water twice a week.

After two months, plants were harvested. The shoot and root elongation, the number of leaves and the shoot and root fresh weights were measured to evaluate growth. Half of plants harvested were quickly frozen in liquid nitrogen, and stored at −80 °C for enzyme assays and gene expression. The rest of plant material was dried at 80 °C until constant weight for metal and nutrient determination.

### 2.2. Chlorophyll Fluorescence and Gas Exchange

Chlorophyll fluorescence was measured in random primary branches (*n* = 10) using a portable modulated fluorimeter (FMS-2, Hansatech Instrument Ltd., England). Dark and light-adapted fluorescence parameters were measured at midday (1500 µmol m^−2^ s^−1^). The minimal fluorescence level in the dark-adapted state (F0) was measured using a modulated pulse (<0.05 µmol m^−2^ s^−1^ for 1.8 μs), and was not sufficient to induce significant physiological changes in the plant. The data stored were an average taken over a 1.6-s period. Maximal fluorescence in this state (Fm) was measured after applying a saturating actinic light pulse of 15,000 µmol m^−2^ s^−1^ for 0.7 s. The value of Fm was recorded as the highest average of two consecutive points. The same leaf section of each plant was used to measure light-adapted parameters. Steady state fluorescence yield (Fs) was recorded after adapting plants to ambient light conditions for 30 min. A saturating actinic light pulse of 15,000 µmol m^−2^ s^−1^ for 0.7 s was then used to produce the maximum fluorescence yield (F′m) by temporarily inhibiting photosystem II (PSII) photochemistry. Using fluorescence parameters determined in both light- and dark-adapted states, the following parameters were calculated: maximum quantum efficiency of PSII photochemistry (Fv/ Fm = (Fm − F0)/ Fm) and quantum efficiency of PSII (ΦPSII = (F′m− Fs)/F′m) [42].

At the same time of chlorophyll fluorescence analysis, gas exchange measurements were taken on random primary branches of each plant (*n* = 10) using an infrared gas analyzer (IRGA) in an open system (LI-6400XT, LI-COR Inc., Neb., USA) equipped with a light leaf chamber (Li-6400-02B, Li-Cor Inc.). Net photosynthetic rate (A_N_), stomatal conductance (g_s_), and intercellular CO_2_ concentration (C_i_) were all determined at ambient CO_2_ concentration, temperature of 25–28 °C, 50 ± 5 % relative humidity, and a photosynthetic photon flux density (PPFD) of 1000 µmol m^−2^ s^−1^ to record each measurement. Photosynthetic area was approximated as half the area of the cylindrical branches, as only the upper half received the unilateral illumination in the leaf chamber [43].

### 2.3. Determination of Nutrient and Metal Content in Plants

The determination of nutrient and metal contents in plant tissues (shoots and roots) was performed by ICP-OES from samples of 1 g dried material at the Ionomics Service of the CEBAS, CSIC (Murcia, Spain).

### 2.4. Antioxidant Enzymes Determination

Enzyme extraction was performed at 4 °C. For that, 0.5 g fresh root tissues were ground and homogenized in 8 mL of 50 mM sodium phosphate buffer (pH 7.6) with 0.1 mM Na-EDTA on ice. The homogenate was centrifuged at 9000 rpm for 20 min at 4 °C. The supernatant was used in enzymatic analyses.

Superoxide dismutase (SOD) was evaluated by monitoring the pyrogallol disappearance spectrophotometrically at 325 nm for 2 min [44]. The reaction mixture contained 640 µL assay buffer (50 mM potassium phosphate, pH 7), 360 µL of ultra-pure water, and 10 µL of plant extract. The reaction was started with the addition of 80 µL of pyrogallol (3 mM). For catalase (CAT), the reaction mixture consisted of 890 µL of extract buffer (50 mM potassium phosphate, pH 7.6) and 100 µL of vegetal extract. The reaction started with the addition of 10 µL 15 % H_2_O_2_. Activity was measured as the decrease in the absorbance at 240 nm [44]. For ascorbate peroxidase (APX) activity [44], the reaction mixture contained 895 µL assay buffer, 100 µL of vegetal extract, and 2.72 µL of 2 mM H_2_O_2_. The reaction started with the addition of 2.5 µL of 0.1 M ascorbate. Activity was recorded as the decrease in absorbance at 290 nm. The reaction of guaiacol peroxidase (GPX) contained 800 µL of solution A (5.9 mL of assay buffer, 100 µL of vegetal extract, and 2 mL of 50-mM H_2_O_2_). The reaction was initiated with the addition of 200 µL of 20 mM guaiacol. The absorbance increase in absorbance was monitored for 2 min at 470 nm [44]. All the enzymatic activities were expressed as specific activity in relation to the protein content of the extract, in mU mg^−1^ protein, upon application of the formula:

$$\mathrm{Specific}\;\mathrm{activity}=\frac{\mathrm{micromole}\;\mathrm{of}\;\mathrm{product}\;\mathrm{formed}\;\mathrm{or}\;\mathrm{micromole}\;\mathrm{of}\;\mathrm{substrate}\;\mathrm{consumed}}{\min.\mathrm{volume}\;\mathrm{of}\;\mathrm{crude}\;\mathrm{extract}\;(\mathrm{mL}).\mathrm{concentration}\;\mathrm{of}\;\mathrm{proteins}\;(\mathrm{mg}\;{\mathrm{mL}}^{-1})}\times 1000$$

### 2.5. Global Stress Evaluation: Oxidative Stress Index (OSI)

Oxidative stress index or OSI is a parameter recently introduced [34,45] to express the extent of the oxidative stress and it is calculated as the average of the prorated values of the different antioxidant enzymes in reference to their values in the control situation, as follows:

OSI=([SOD]/[SOD]0 + [CAT]/[CAT]0 + [APX]/[APX]0 + [GPX]/[GPX]0)/4

where [SOD], [CAT], [APX], and [GPX] are the values of these enzymes in the presence of heavy metals (with or without bacteria). [SOD]0, [CAT]0, [APX]0, and [GPX]0 were the control values, i.e., in the absence of both metals and bacteria. A value higher than 1 expressed that the roots were stressed, whereas values lower than 1 corresponded to roots without oxidative stress.

### 2.6. Isolation of Plant RNA and qRT-PCR of Stress Related Genes

Total RNA (two independent extractions for each sample) was extracted from 100 mg root tissue of *M. sativa* grown under different metal /inoculation treatments, using IQeasyTm Plant RNA extraction following the manufacturer instructions. To ensure that there was no residual DNA in RNA preparations, they were additionally treated with DNase (ThermoFisher, USA). Immediately after extraction, all samples were subjected to cDNA synthesis using the QuantiTec Reverse Transcription Kit (Qiagen, Hilden, Germany) according to the manufacturer instructions. cDNA samples were stored at −80 °C for up one week. The qRT-PCRs were performed using RealMODTM Probe SF2X qPRC mix and an ECO thermocycler (Illumina) following the supplier’s instructions. Table 1 shows the primer pairs used for each gene and the Tm used in PCR amplification reactions. The amplification conditions were: initial denaturation at 95 °C for 2 min, 40–50 cycles at 95 °C for 5s, 48–66 °C (depending on the gene) for 10 s, 72 °C for 15 s, and a final step at 95 °C 15 s, 55 °C for 15 s, and 95 °C for 15 s. The housekeeping gene His1 was used to normalize results from different samples. Expression signals were quantified and normalized using EcoTM Software version 4.1.2.0 (Illumina). The expression fold was calculated according to [46]:

ΔCq = AVECq(TargetAssay) – AVECq(ReferenceAssay)
ΔΔCq = ΔCq(TestSample) − ΔCq(ReferenceSample)
RQ = 2 − ΔΔCq

### 2.7. Observation of Biofilms by Scanning Electron Microscopy

The formation of biofilms onto the plant surface was observed by SEM. For that, plants were cultivated in square plates (12 × 12 cm) filled with plant medium consisting of B&D medium [47] and plant agar (15 g L^−1^). Seeds were disinfected as before and germinated in petri dishes onto wet filter paper for 2 days in the dark. Germinated seed were transferred to square plates containing the agar–B&D medium without or with metals (25 µM Cu, 100 µM Zn, and 100 µM Pb). Three plates for each condition were inoculated (each seed was inoculated with 100 µL of an overnight culture of each strain or the mixture of all of them for the consortium). The plates were sealed, covered outside with black paper until the line of the seeds in order to keep roots from light, and incubated at 25 °C /15 °C with 16 h/8 h dark/light photoperiod for 20 days. After this time, plants were harvested and roots separated and washed three times with sterile distilled water. Pieces of roots of approximately 2–3 mm were fixed with 2.5% glutaraldehyde dissolved in 0.2 M cacodilate buffer pH 7.2 for 3 h at room temperature. After washing three times with 0.2 M cacodilate buffer pH 7.2, root samples were dehydrated in acetone series (50% to 100%) and dried using the critical point drier Leica EM CPD300 at 31 °C and 73.8 bar, sputtered with Au-Pd (10 nm), and observed with a scanning electron microscope Jeol 6450LV at the Microscopy Service of the University of Sevilla, Spain.

### 2.8. Statistical Analysis

Results are means ± SE (standard errors) of 10 determinations for growth parameters, three for mineral nutrients and metal accumulation, five for enzymatic activity, and six for gene relative expression (two independent samples × three replicates). Differences among treatments were assessed by one-way ANOVA; the averages were compared by the Student, Newmann, Keuls (SNK) test. Significant differences at *p* < 0.05 are indicated by different letters.

## 3. Results

### 3.1. Plant Growth Parameters

**Figure 1:**
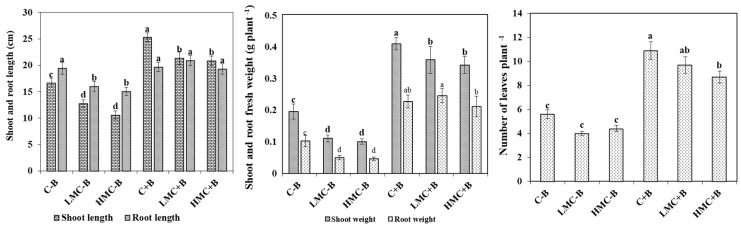
Shoot, root length, and weight and number of leaves of *Medicago sativa* plants subjected to different treatments. Means (±SE) within the same graph followed by different letters are significantly different at *p* < 0.05. C-B: control without inoculation; LMC-B: low metal concentrations, without inoculation; HMC-B: high metal concentrations, without inoculation; C+B: control inoculated with bacteria consortium; LMC+B: low metal concentrations, inoculated with bacteria consortium; HMC+B: high metal concentrations, inoculated with bacteria consortium.

The effect of heavy metals and/or inoculation with bacteria on the growth of *M. sativa* is illustrated in Figure 1. Results show that cultivation of plants in the presence of heavy metals negatively affected the growth parameters, such as shoot and root lengths and weights, as well as the number of leaves. Moreover, heavy metal stress had significant deleterious effects, causing toxic symptoms in plants shoots like chlorosis and necrosis (not shown). The highest concentration of heavy metals (HMC) was the most stressful and had the strongest consequences on plant biomass of both shoots and roots (36% and 48% reduction in case of shoot length and weight, and 22% and 54% in the case of roots, respectively). However, inoculation of alfalfa with the bacterial consortium had a significant effect at *p* < 0.05. The inoculation clearly promoted plant growth in the absence of metals. The PGPR activity was the most evident in case of C+B treatment compared to non-inoculated treatment (C-B); for instance, the length of shoots increased between 22% and 77%, whereas shoot fresh weight rose up to 220% as compared to non-inoculated plants. Moreover, upon metal exposure, the inoculation treatment alleviated the stress; this was evident by the enhancement of growth and the absence of stress symptoms. Only in the highest metal concentration (HMC) were significant diminutions (10–15%) of shoot and root lengths and weights observed, while the inoculation treatment seemed to fully protect plants under the low metal level (LMC). It should be noted that nodules appeared on the roots of the plants (Figure S1) but, since we inoculated all the four strains in consortium, the nodule occupancy by RhLO6 or RhLO8 or both can not be established.

### 3.2. Physiological State of The Plants

**Figure 2:**
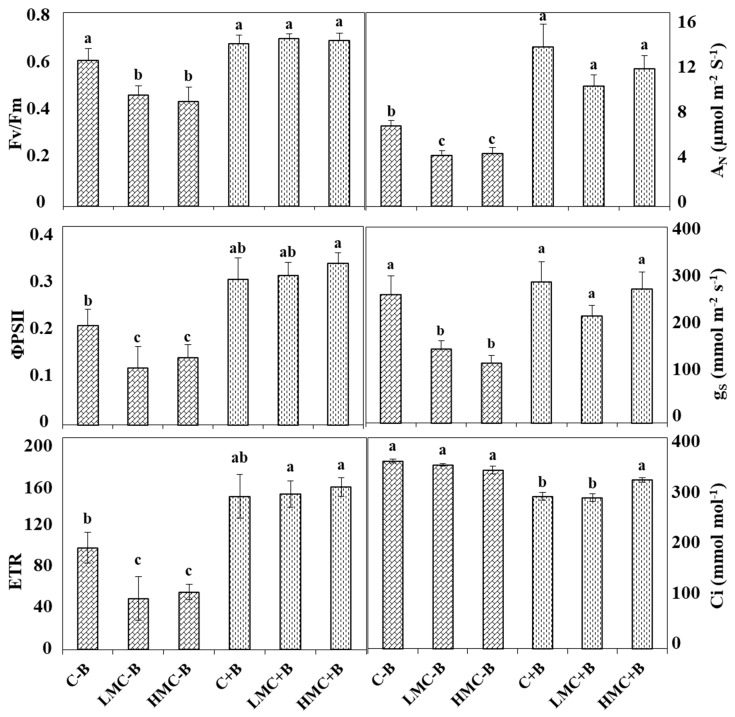
Effect of heavy metals and inoculation treatments (details are in Materials and Methods) on maximum quantum efficiency of PSII (Fv/Fm); quantum efficiency of PSII (ΦPSII); electron transport rate (ETR); net photosynthetic rate (A_N_); stomatal conductance (g_s_); and intercellular CO_2_ concentration (C_i_), in random primary branches of *Medicago sativa* plants. Values are means ± S.E. (*n* = 10). Different letters indicate means that are significantly different from each other (*p* < 0.05). C-B: control without inoculation, LMC-B: low metal concentrations, without inoculation; HMC-B: high metal concentrations, without inoculation; C+B: control inoculated with bacteria consortium; LMC+B: low metal concentrations, inoculated with bacteria consortium; HMC+B: high metal concentrations, inoculated with bacteria consortium.

Regarding the effect of inoculation on *M. sativa* photosynthetic rate, our results revealed that maximum quantum efficiency of PSII photochemistry (Fv/Fm) and quantum efficiency of PSII (ΦPSII) were greater in plants inoculated with the bacterial consortium. In all cases (Control, LMC, and HMC), inoculation with bacteria led to an increment of 11–57% and 46–162% for Fv/Fm and ΦPSII respectively, with the most apparent effect in plants under metal stress. In addition, the electron transport rate (ETR) was lower in non-inoculated plants than in the inoculated ones; increments between 50% and 200% were observed upon inoculation, with significant differences between treatments (one-way ANOVA, *p* < 0.05; Figure 2). There was a relationship between net photosynthetic rates (A_N_) and heavy metal concentration and inoculation. Cultivation of plants under heavy metals stress reduced the A_N_ compared to the control. However, the inoculation with the bacterial consortium raised plant photosynthetic rates. In the same way, the stomatal conductance (g*_s_*) was also negatively affected by the metal concentration in non-inoculated plants, whereas improvements of 9% (control), 44% (LMC), and 122% (HMC) were observed in plants inoculated with the bacterial consortium compared to non-inoculated plants. The intercellular CO_2_ concentration (C_i_) showed the opposite pattern, since C_i_ was always higher in non-inoculated plants, probably reflecting lower rates of C fixation. This situation was reverted by inoculation with the metallo-resistant strains which significantly diminished C_i_ values. In general, an overall conclusion is that metals diminished all the physiological parameters in non-inoculated plants, but inoculated plants maintained their physiological state.

### 3.3. Nutrient Composition of Plants

The effect of metals and inoculation with metallo-resistant bacteria on nutrient acquisition has been studied. Results are shown in Table 2. Phosphorus metabolism seemed to be affected by inoculation, since the inoculated plants registered significantly higher content of phosphorus than the non-inoculated ones, with constant trend. Other element such as sodium showed higher values in inoculated plants. The rest of the elements showed variable differences between inoculated and non-inoculated plants, but without a clear trend.

### 3.4. Determination of Antioxidant Enzymes

**Figure 3:**
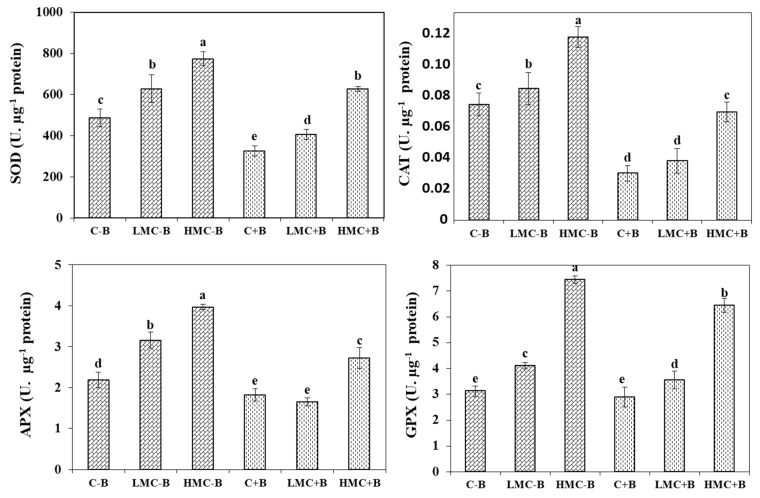
Enzyme activity of superoxide dismutase (SOD), catalase (CAT), ascorbate peroxidase (APX), and guaiacol peroxidase (GPX) in roots plants grown under different treatments. Data are means ± SE of five determinations. Within the same graph, different letters indicate significant differences at *p* < 0.05. C-B: control without inoculation; LMC-B: low metal concentrations, without inoculation; HMC-B: high metal concentrations, without inoculation; C+B: control inoculated with bacteria consortium; LMC+B: low metals concentrations, inoculated with bacteria consortium; HMC+B: high metal concentrations, inoculated with bacteria consortium.

In order to understand antioxidant enzymes involved in management of the oxidative stress caused by heavy metals, the activities of SOD, CAT, APX, and GPX were evaluated (Figure 3). Increasing concentrations of heavy metals significantly induced enzyme activity of *M. sativa* compared to the control in both inoculated and non-inoculated treatment. SOD activity seemed to be the enzyme with the highest involvement in stress management, since the values of this enzyme always increased by 25–93% while CAT was enhanced by 44–82%, whereas APX activity did not increase for some treatments (LMC). Moreover, the inoculation with the bacterial consortium led to a reduction of the activities of ROS-scavenging enzymes in *M. sativa* roots. Treatment with this consortium reduced GPX activity by 9–13%. The largest reduction of enzymatic activity was noticed in the cases of SOD (40–67%) and CAT (31–57%). On its side, APX activity registered decreases of 48% and 31% at LMC and HMC as compared to the corresponding non-inoculation treatments. In general, it could be said that inoculation with bacteria kept the levels of ROS-scavenging enzymes below the level of non-exposed plants at the low metal concentration (LMC). Only at the highest concentration of metals (HMC) did the levels of some enzymes increase, although they always remained below those of non-inoculated plants.

### 3.5. Evaluation of the Overall Stress: The Oxidative Stress Index (OSI)

**Figure 4:**
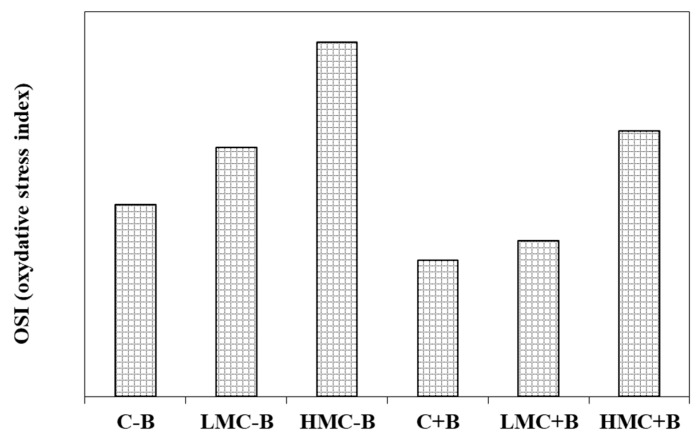
Oxidative stress index (OSI) in roots of plants grown under different treatment. C-B: control without inoculation; LMC-B: low metal concentrations, without inoculation; HMC-B: high metal concentrations, without inoculation; C+B: control inoculated with the bacterial consortium; LMC+B: low metals concentrations, inoculated with bacteria consortium; HMC+B: high metal concentrations, inoculated with the bacterial consortium.

To estimate the overall oxidative stress, the OSI was proposed [34,45]. The high value of OSI was recorded in non-inoculated plants grown at HMC, for which the OSI was almost twice than the control. The inoculation with the consortium reduced the value of OSI below 1 for both the control (without metal supplementation) and LMC, values that are considered to indicate non-stressed plants, whereas at the highest metal concentration (HMC), the value of OSI was higher than 1 (Figure 4).

### 3.6. Expression of Stress-Related Genes

In order to understand the molecular mechanisms underlying plants root protection by bacteria, expression levels of several stress related genes were investigated. Based on their involvement in metal stress management, nine genes were selected which were representative of the different pathways for stress alleviation [34] and their involvement in bacteria-plant symbiosis [39] (Table 1), as follows: Histone 1 (*His1*) was used as the housekeeping gene; *NRAMP1* (natural resistance-associated macrophage protein) is a Mn transporter which is known to be overexpressed under As and Cd stress in rice [48,49]; glutathione reductase (*GR*) is involved in the maintenance of the redox state [36]; and phytochelatin synthase (*PCS*) is involved in the synthesis of phytochelatins for metal complexation [50]. The gene *CP450* coding for cytochrome P450 is involved in stress management under xenobiotics and metal exposure [51,52]. The gene coding for phenylalanine ammonia lyase (*PAL*) is involved in secondary metabolism and lignin synthesis, which is induced by metals [34,53,54] and the receptor of ethylene (*ETR1*) the plant hormone more related to stress situations [55].

**Figure 5:**
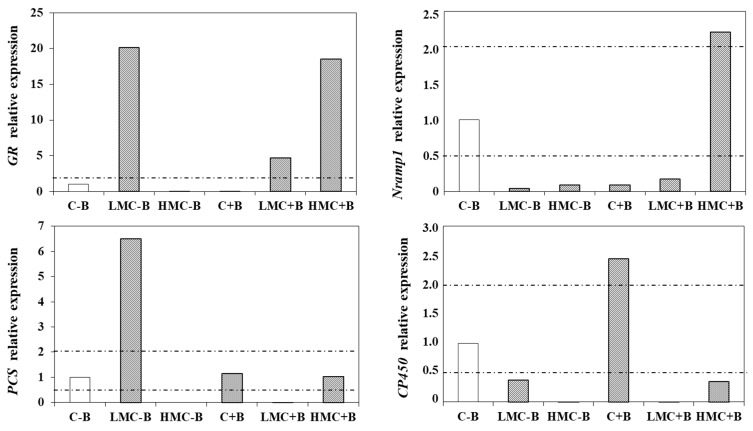
Expression of glutathione reductase (*GR*), natural resistance-associated macrophage protein transporters (*NRAMP1*), phytochelatin synthase (*PCS*), and cytochrome P450 (*CP450*) in roots of plant grown under different concentrations of metals and/or inoculation treatments. For each gene, the expression level is relative to that of control plants (without metals and non-inoculated) considered as 1. Expression levels were calculated using *His1* as the housekeeping gene. Dashed lines indicate expression between 0.5 and 2, considered as not significantly different in qT-PCR, since changes in gene expression would correspond to less than one cycle amplification. C-B: control without inoculation; LMC-B: low metal concentrations, without inoculation; HMC-B: high metal concentrations, without inoculation; C+B: control inoculated with the bacterial consortium; LMC+B: low metals concentrations, inoculated with the bacterial consortium; HMC+B: high metal concentrations, inoculated with the bacterial consortium.

In the first place, it has to be noted that the quality of RNA obtained from samples of roots in the highest metal concentration HMC and non-inoculated was insufficient and amplification of any gene was observed, even the housekeeping gene, so they were discarded. This could be also interpreted as the highest stress in these plants. The results of relative gene expression are illustrated in Figure 5. Besides *His1*, four genes gave amplification signals, in particular *NRAMP1*, *GR*, *PS,* and *CP450*, whereas *PAL* and *ETR1* did not produce any amplification signal or the identity of the PCR product could not be confirmed by sequencing. Since the differences corresponded to less than one PCR cycle, expression levels between 0.5 and 2.0 as compared to the control (plants without metals and without inoculation) were not considered as statistically significant compared with the control (plants with no heavy metal supplementation and no inoculation). In non-inoculated plants, the presence of metals at the lowest concentration LMC led to an enhancement of the expression of *GR* (20-fold) revealing a strong oxidative burst in these plants. The same concentration of metals did not produce an induction of this activity in inoculated plants, in which overexpression was only measured at the highest metal concentration HMC. Regarding *PCS*, a 6.5-fold induction was observed in non-inoculated plants, reflecting metal detoxification through complexation and vacuole compartmentalization. However, in inoculated plants, this induction was abolished. The Mn transporter *NRAMP1* was repressed by metals in non-inoculated plants, maybe as a mean to block toxic metal uptake. By contrast, an enhancement of 2.2-fold was registered in plants grown at the highest metal concentration HMC upon inoculation. In addition, the gene *CP450* was induced, more than by metals, by the inoculation treatment (2.44-fold enhancement) even in the absence of metals.

### 3.7. Metal Accumulation in Plants

**Figure 6:**
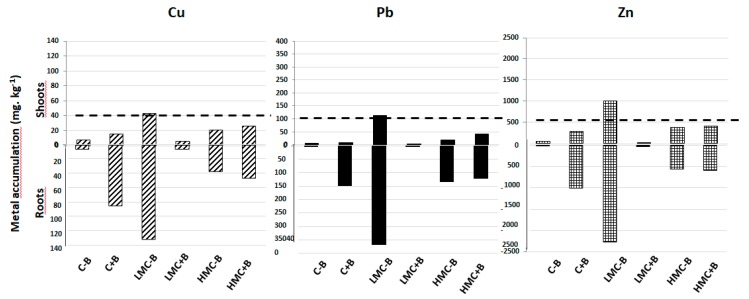
Metal accumulation in shoots and roots of *Medicago sativa* plants in the presence of different metal concentrations and/or inoculation treatments. C-B: Control without inoculation; LMC-B: low metal concentrations, without inoculation; HMC-B: high metal concentrations, without inoculation; C+B: control inoculated with the bacterial consortium; LMC+B: low metal concentrations, inoculated with the bacterial consortium; HMC+B: high metal concentrations, inoculated with the bacterial consortium. Dashed lines represent the threshold for animal grazing in aboveground tissue [56].

The accumulation of metal in plants is shown in Figure 6. Alfalfa is a non-hyperaccumulating plant, so metal is mainly accumulated in roots, with low translocation to shoots. As the external concentration of metals increased, so did the accumulation of metals in both shoots and in roots. At the highest metal concentration (HMC), the accumulation of Cu and Pb reached the threshold recommended for animal grazing, whereas Zn accumulation surpassed this limit. In all cases, the inoculation with bacterial consortium led to a diminution of metal accumulation; this decrease was observed both in shoots (reductions between 42% and 65% depending on the metal) and in roots (where the reductions were between 47% and 66% depending on the metal). Moreover, inoculation with the selected strains allowed the safe cultivation of this plant on metal polluted soils, since all the values of metal accumulation in aboveground tissues always remained below the threshold for animal grazing [56].

### 3.8. Colonization of Plant Roots by Bacterial Cells

**Figure 7:**
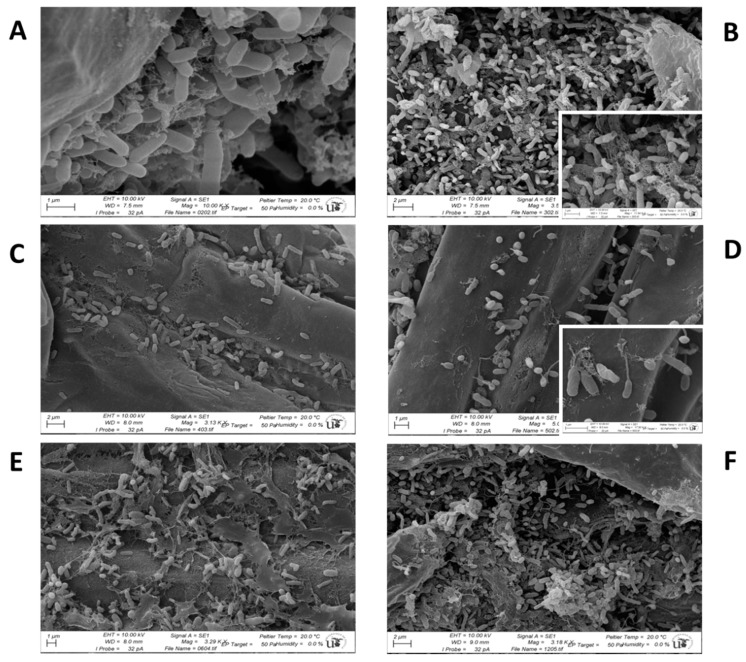
Observation of root colonization by SEM. A. Biofilm formed onto *M. sativa* roots by *Proteus* sp. DSP1 (**A**), *Pseudomonas* sp. DSP17 (**B**), *Ensifer meliloti* RhLO6 (**C**), and *Ensifer meliloti* RhLO8 (**D**) when plants were cultivated in the absence of supplementary metals (note the extracellular polysaccharide fibers produced by the strain DSP17 (Figure 7B, right corner) and the peduncle-like structures formed by the strain RhLO8 (Figure 7B, right corner)). (**E**) Biofilm formed onto *M. sativa* roots by the consortium of the four bacteria when plants were cultivated in the absence of supplementary metals (note the different morphologies: thick roods, thin and long rods, and coccobacilli and the fibers of polysaccharide). (**F**) Biofilm formed onto *M. sativa* roots by the consortium of the four bacteria in plants grown in the presence of metals (note the different morphologies: thick roods, thin and long rods, and coccobacilli, together with the presence of bacteria showing a peduncle-like structure).

The colonization of plant roots by the bacteria was analyzed by SEM (Figure 7). When bacteria were applied individually, it could be seen that the PGPR strains DSP1 and DSP17 showed wide root colonization with extensive colonies which almost covered the whole root surface (Figure 7A,B). In the case of the *Pseudomonas* strain, the presence of a dense web of extracellular material was observed (Figure 7B, detail at the right corner). In the case of the rhizobial strains, root colonization was also observed although not so dense cover as for the PGPR strains (Figure 7C,D). It is noteworthy the formation of some kind of peduncles-like structures or thick fibers by the strain *Ensifer meliloti* RhLO8, together with extracellular thin fibers (Figure 7B, detail at the right corner) which could be involved in the attachment of this strain to the plant surface. The consortium of the four bacteria also showed a dense root cover, in which the distinct bacterial morphologies were observed (Figure 7E). In plants cultivated in the presence of metals and inoculated with the bacterial consortium, a dense carpet of bacteria was also seen, in which the different morphologies could be detected (Figure 7F), indicating that the plant–bacteria interaction was not abolished but even promoted under metal stress. The presence of a dense web of extracellular material in which the bacteria were entrapped was observed, together with “pedunculated“ bacteria probably corresponding to RhLO8.

## 4. Discussion

The effect of rhizobacteria inoculation on growth, physiology, heavy metal uptake, ROS scavenging enzymes, and expression of some related of *M. sativa* plants to heavy metal stress was studied. The obtained results showed that *M. sativa* cultivation under heavy metal stress negatively affected the growth parameters (shoot and root length, fresh weight, and leaf number). The toxicity of metals and metalloids to this plant was previously reported; for instance, it inhibits growth and causes a diminution of nodulation by affecting the number of infections [57]. On their side, metals such as Cd, Cr, Cu, Ni, and Zn caused inhibition of seed germination and of the plant growth [58].

In our work, the diminution of growth in the presence of these toxicants correlated with deep changes in the physiological state of the plants, as deduced from the physiological parameters measured, in particular, maximum quantum efficiency of PSII (Fv/ Fm), quantum efficiency of PSII (ΦPSII), electron transport rate (ETR), net photosynthetic rate (A_N_), stomatal conductance (g_s_), and intercellular CO_2_ concentration (C_i_). The obtained results probed that heavy metals affected plant physiology and reduced the parameters, indicating damage to the photosynthetic machinery. In fact, photosynthesis is one of the processes the most affected by heavy metals such as copper [59] and cadmium [60].

Our results prove that the inoculation of plants with rhizobacteria consortium alleviated the stress caused by heavy metals, enhancing all the growth parameters so that the yield of inoculated plants grown in the presence of metals almost did not show significant differences as compared to unexposed controls. The phytoprotection exerted by bacteria against heavy metal stress correlated with an increment of all physiological parameters recorded with the exception of C_i_. Abundant literature is being produced on the positive effect of inoculation with metallo-resistant bacteria on plants cultivated under metal stress. For instance, cultivation of *Atriplex halimus* and *Arthrocnemum macrostachyum* in sediment polluted with heavy metals showed a reduction in chlorophyll florescence and gas exchange, while inoculation with bacteria improve them [27,34,61]. Multiple mechanisms account for this effect, since bacteria can enhance plant nutrition through phosphate solubilization, iron acquisition, nitrogen fixation, etc. Besides, they can stimulate plant growth through secretion of auxins, or they can reduce plant stress via the ACC-deaminase activity. [62,63,64] There were effects on mineral nutrition; particularly affected were two elements: phosphorus and sodium. In this context, it should be remembered that some of the bacteria displayed phosphate solubilizing ability, perhaps increasing the content of P in plants, both in shoots and roots. It would be interesting to determine the salt tolerance of the bacteria since, having being isolated from a desert area, it is possible that they could display drought and salt tolerance and could be the explanation for the increase in Na content in inoculated roots [65,66].

In order to counterbalance the production of ROS due to metal exposure, plants synthesize non-enzymatic metabolites (ascorbate, glutathione and polyphenols) and enzymes (CAT, SOD, GPX and APX) under metal stress condition [34,36]. Some studies reported that rhizobacteria inoculation can reduce the stress of plants grown on sediments with different physicochemical properties [20,22,27,37,65]. Nonetheless, *Arthrocnemum macrostachyum* inoculation with bacteria consortium from the endosphere showed less significant effects in comparison with bacteria consortium from the rhizosphere [67]. The mechanisms underlying this protective effect are being deciphered. Cultivation of plants under metal stress increased the levels of ROS-scavenging enzymes SOD, CAT, APX, and GPX, while the inoculation with bacteria decreased these parameters [27,34]. The lower levels of OSI (a parameter introduced to get an overall idea of the oxidative stress) indicated that rhizobacteria can fully mitigated the oxidative stress induced by heavy metals [34].

In a second approach, expression levels of nine genes related to heavy metal stress management and nodulation symbiosis were investigated. Four genes were amplified with our primers and conditions. In our hands, the genes amplified were *NRAMP1, GR, PCS,* and *CP450*. The rest of the genes did not give any PCR product upon using DNA as the template or the product was not confirmed by sequencing. Since the primers were designed on consensus sequences based on the sequences of several grasses available in the database [34], it is possible that the same sequences are not very conserved in legumes or maybe the amplification conditions have to be optimized for each particular gene. In our case we used the same conditions as in [34]. Our results suggested that gene expression depends on the concentration of heavy metals and the inoculation treatment. Genome-wide expression analysis suggested variable number of genes differentially expressed in root in response to As(V), Cd, Pb, and Cr(VI) stresses. [68] The induction of the phytochelatins synthesis pathways is evident by the enhanced expression of the gen *PCS*. Complexation of metals with phytochelatins and storage of the complexes into the vacuole is the main detoxification mechanism is non-hyperaccumulating plants as it is the case of alfalfa [17,39,50]. Moreover, glutathione, besides being the substrate for the synthesis of phytochelatins, plays a fundamental role in management of the stress produced by metal exposure and hence the induction of *GR* in order to keep a pool of reduced glutathione [36,37]. Both genes have differential expression in the presence or the absence of bacteria: whereas *GR* expression seems to be enhanced by the bacteria, probably reflecting the better management of the stress in inoculated plants, *PCS* expression was higher in non-inoculated plants, maybe due to the lower level of metals accumulated in inoculated plants with regard to the non-inoculated partners. Regarding the gene *MRAMP1*, the regulation by metals is known [48,49]. In our case, the inoculation treatment seems to enhance this gene, particularly at the highest metal concentrations. In contrast, the gene *CP540* was specifically enhanced by the presence of bacteria, even in the absence of metals. This could be interpreted as a defense mechanism of the plants towards the bacteria. The activation of genes involved in biotic stress has been reported at the first stages of some symbiotic processes such as nodulation of legumes by rhizobial strains [39,69,70].

One of the most important points in this study is the diminution of metal accumulation in alfalfa plants upon inoculation. This is a metal excluder plant [17,58] which accumulates metals preferably in roots, the inoculation treatment even decreased metal translocation to shoots, since all the values of metal concentrations fell far below the threshold for animal grazing. Similar results have been reported recently. For instance, inoculation of *Spartina densiflora* with gram-negative bacteria led to lower levels of metal accumulation [71]. In this particular, all the bacteria used in our consortium are gram negative, some of them producing a great amount of exoplysaccharide (EPS) which is the case of the rhizobial strains such as *Ensifer meliloti* RhLO6 and RhLO8. These EPSs are specific signals in the rhizobium–legume symbiosis interaction [72,73,74]. It is known that metals, positively charged, can be bound to negative charges in EPS (biosorption) through electrostatic interactions [75,76]. Thus, the formation of a dense biofilm onto the root, as we could observe by SEM, may be a barrier to the entrance of metals into the plant root. All four strains have the ability to form this biofilm, in spite that the PGPR strains *Proteus* sp. DSP1 and *Pseudomonas* sp. DSP17 formed a more dense structure as compared to the rhizobial strains. In consortium, the different morphologies could be observed. The formation of a “peduncle-like” structure by the strain *Ensifer meliloti* RhLO8 is outstanding. In our hands, this kind of structure has been only observed when the bacteria are attached to plant roots and we could not detect this peduncle in previous SEM on glass surfaces (Raklami et al., accompanying paper), so it could be suggested that the structure has a biological function. It is known that some rhizobial strains display pili-like or needle-like structures when infecting legumes, not via root hair curling, but via crack entry or intercellular entry [77,78]. These pili are part of a type III secretion system harbored by both symbiotic and pathogenic bacteria [79,80]. However, these pili have a much thinner diameter, around 6–8 nm [77]. In our case the peduncle-like structure can have more between 100–200 nm, as observed by SEM. We do not know whether this structure could be a mechanism of rhizobial strains to infect legumes or another kind of structure aimed to attach the bacteria to the plant surface, since it is not produced upon attachment to glass surfaces. This aspect deserves further investigation. In any case, it could be noted that growing plants on agar medium could magnify the colonization of roots by the bacterial strains, and in this sense, semihydroponic cultures or inert substrates such as perlite or vermiculite could provide a more realistic approach to the colonizing ability of the strains.

In our case, it was demonstrated that the dense bacterial biofilm was not abolished, but even promoted, in the presence of metals. In any case, the bacterial biofilm seems to be a barrier for metal entrance into the plant root, thus protecting plants from metal stress and allowing safe cultivation of this plant in moderately polluted soils, since the concentrations of metals in aboveground tissues are always below the maximum recommended for animal grazing [56].

## Figures and Tables

**Table 1:** Primers pairs and conditions for qRT-PCR amplification of stress related genes, nodulation genes, and housekeeping genes. The sequences of the primers used for the amplification of the genes *His1, GR, NRAMP1, PS, CP450* and *PAL* were already published [34], while the sequences of the genes *ETR, CHS4, LYSM* and *ENOD2* were reported in [39].

Gene	Name	Tm	Primer Sequence 5’-3’	Expected Band Size	Amplification
***His1***	Histone 1	63 °C	FW:5’AGACCACCAAGTACTACTGCAC 3’ RV:5’ ATACCAGCCCTCAAACCACCA 3’	150 bp	**Positive**
***GR***	Glutathione reductase	63 °C	FW:5’ GTGCTTCGTGGATGTGTTCCAAAG 3’ RV:5’ GTGCTCCAGTCATGCTTCGGATCAG 3’	121 bp	**Positive**
***NRAMP1***	Natural resistance-associated macrophage proteins transporters	57 °C	FW:5’ GTTATGCCGCACAATCTTTTC 3’ RV:5’ AGAGCCAATCCTCTTTCTCTATC 3’	119 bp	**Positive**
***PS***	Phtochelatin synthase	56 °C	FW:5’ TTTCAAGTATCCTCCTCACTGGGTTC 3’ RV:5’ TTCATCTTTACARCTCACAGTAT 3’	154 pb	**Positive**
***CP450***	Cytochrome P450	56 °C	FW:5’AAAGAAGTGTTGAGGCTGCA 3’ RV:5’ ATAGCCCACATGTTGACCAT 3’	128 pb	**Positive**
***PAL***	Phenylalanine ammonia lyase	66 °C	FW:5’ GAAGGTGGACGCCGCCGAGGC 3’ RV:5’ GAGCCCACGGAGGTGCCAT 3’	108 pb	Negative
***ETR***	Ethylene receptor	56 °C	FW:5’ GVTGTTGCTCTTCTCATGC 3’ RV:5’ TGATTCATGACAGCYAGAAAATC 3’	116 pb	Negative
***CHS4***	Chalcone synthase	59 °C	FW:5’ TCAGCTCAAGATGGATTGAAGA 3’ RV: 5’ GCCAATTAACACACCCCATT 3’	-	Negative
***LYSM***	Receptor of nod factor	48 °C	FW:5’ TCTAGTCAACTCCAGCATGGTC 3’ RV:5’ CCTTGGAGAAACAACAGTAGTAGACTC 3’	-	Negative
***ENOD2***	Early nodulin 2	64 °C	FW:5’ CGACCACATGTGCATCCACCGGCC 3’ RV:5’ CGGGTTTCTCATGAGGTGGTTGG 3’	-	Negative

Positive: The gene was amplified (confirmed by sequencing). Negative: no amplification was achieved (using DNA as the template) under these conditions or the product identity was not confirmed by sequencing.

**Table 2:** Concentration of mineral nutrients in shoots and roots of alfalfa plants under different heavy metals and/or inoculation treatments. Data are means ± standard deviations of three independent samples.

SAMPLE	TISSUE	Ca (g/100g)	K (g/100g)	Mg (g/100g)	Na (g/100g)	P (g/100g)	S (g/100g)
C-B	SHOOT	2.03 ± 0.03	2.58 ± 0.04	0.29 ± 0.01	0.07 ± 0.01	0.20 ± 0.03	0.63 ± 0.04
	ROOT	0.19 ± 0.02	1.34 ± 0.02	0.16 ± 0.02	0.06 ± 0.01	0.23 ± 0.02	0.23 ± 0.02
LMC-B	SHOOT	1.38 ± 0.02	2.69 ± 0.02	0.36 ± 0.03	0.12 ± 0.02	0.22 ± 0.02	0.48 ± 0.02
	ROOT	0.59 ± 0.01	3.39 ± 0.05	0.36 ± 0.01	0.16 ± 0.02	0.40 ± 0.03	0.58 ± 0.05
HMC-B	SHOOT	1.94 ± 0.02	2.42 ± 0.01	0.33 ± 0.02	0.07 ± 0.01	0.19 ± 0.01	0.57 ± 0.03
	ROOT	0.37 ± 0.03	1.73 ± 0.02	0.34 ± 0.02	0.15 ± 0.02	0.27 ± 0.03	0.44 ± 0.02
C+B	SHOOT	1.37 ± 0.02	2.09 ± 0.01	0.22 ± 0.02	0.13 ± 0.02	0.55 ± 0.03	0.38 ± 0.01
	ROOT	0.30 ± 0.01	0.95 ± 0.02	0.22 ± 0.01	0.39 ± 0.03	0.49 ± 0.04	0.22 ± 0.04
LMC+B	SHOOT	1.33 ± 0.02	2.59 ± 0.03	0.34 ± 0.02	0.51 ± 0.02	0.33 ± 0.02	0.41 ± 0.02
	ROOT	0.21 ± 0.02	1.70 ± 0.02	0.17 ± 0.01	0.32 ± 0.03	0.47 ± 0.01	0.24 ± 0.03
LMC+B	SHOOT	2.00 ± 0.03	4.00 ± 0.02	0.45 ± 0.03	0.42 ± 0.04	0.45 ± 0.02	0.64 ± 0.03
	ROOT	0.28 ± 0.02	1.88 ± 0.01	0.21 ± 0.02	0.38 ± 0.01	0.57 ± 0.03	0.31 ± 0.04

## Supplementary Materials

The following are available online at https://www.mdpi.com/2076-2607/7/7/212/s1.
