# Supplementary material for: Safe Cultivation of Medicago sativa in Metal-Polluted Soils from Semi-Arid Regions Assisted by Heat- and Metallo-Resistant PGPR
Source: Microorganisms. 2019 Jul 22;7(7):212. doi: 10.3390/microorganisms7070212 (PMC6680742; doi:10.3390/microorganisms7070212)
Supplement: Supplementary file 1 [file microorganisms-07-00212-s001.pdf]

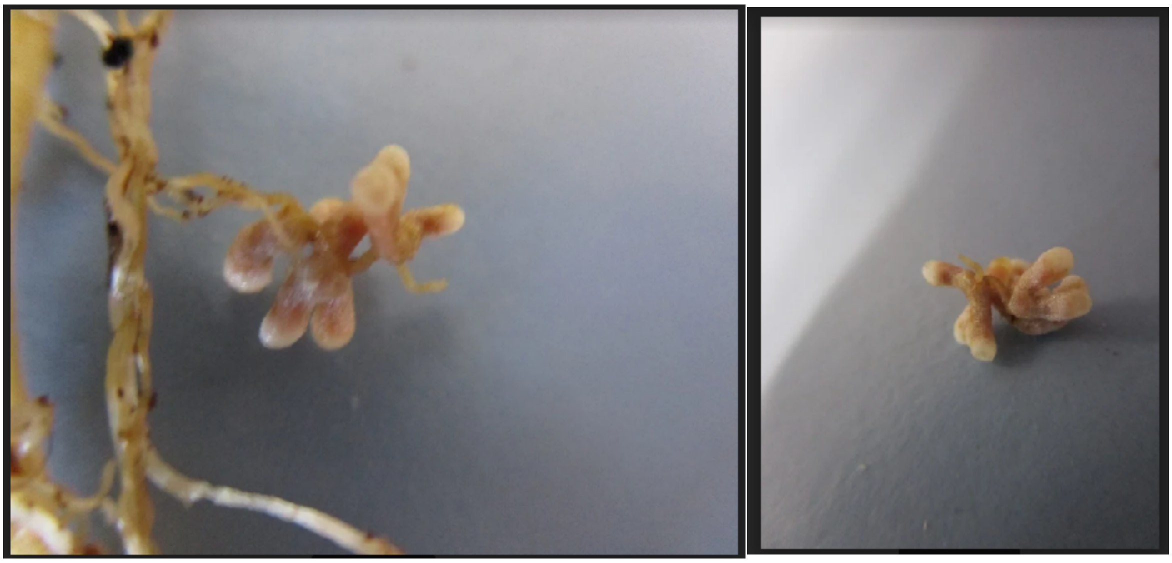

(**a**) (**b**)

Figure S1. (**A**) Nodules formed on alfalfa roots by the consortium of bacteria. Since all the strains were inoculated together, the occupancy by one of the rhizobial strains RhLO6 or RhLO8 can not be established. (**B**) Nodules were lobulated and pink, indicative of nitrogen Fixation.
